# Hypogonadism is frequent in very old men with multimorbidity and is associated with anemia and sarcopenia

**DOI:** 10.1007/s00391-023-02235-7

**Published:** 2023-09-06

**Authors:** Sabine Schluessel, Martin Bidlingmaier, Sebastian Martini, Martin Reincke, Nicole Reisch, Anna Schaupp, Günter Stalla, Daniel Teupser, Ralf Schmidmaier, Michael Drey

**Affiliations:** 1 Medizinische Klinik und Poliklinik IV, Department of Geriatrics, LMU Klinikum, Ludwig-Maximilians-Universität München, Munich, Germany; 2 Medizinische Klinik und Poliklinik IV, Department of Endocrinology, LMU Klinikum, Ludwig-Maximilians-Universität München, Munich, Germany; 3 Medicover Neuroendocrinology, Munich, Germany; 4 Institute of Laboratory Medicine, LMU Klinikum, Ludwig-Maximilians-Universität München, Munich, Germany; 5 Ziemssenstraße 5, 80336 Munich, Germany

**Keywords:** Testosterone, Osteoporosis, Geriatrics, Dual-energy X-ray absorptiometry, Primary and secondary hypogonadism, Testosteron, Osteoporose, Geriatrie, Dual-Röntgen-Absorptiometrie, Primärer und sekundärer Hypogonadismus

## Abstract

**Background:**

Clinical data regarding hypogonadism in very old men with multimorbidity are rare. Hypogonadism can contribute to osteoporosis, anemia and sarcopenia and is therefore a relevant problem for geriatric patients.

**Methods:**

A total of 167 men aged 65–96 years (mean 81 ± 7 years) admitted to an acute geriatric ward were included in a cross-sectional study. Body composition derived from dual-energy X‑ray absorptiometry, bone mineral density, handgrip strength, multimorbidity, polypharmacy and laboratory values were obtained from the routine electronic clinical patient file.

**Results:**

Hypogonadism was present in 62% (*n* = 104) of the study participants, of whom 83% showed clinical manifestation of hypogonadism (hypogonadism in combination with anemia, sarcopenia and/or low T‑score). The subgroups showed a distribution of 52% primary and 48% secondary hypogonadism. Compared to the eugonadal patients, hypogonadal patients had reduced handgrip strength (*p* = 0.031) and lower hemoglobin levels (*p* = 0.043), even after adjustment for age, body mass index and glomerular filtration rate.

**Conclusion:**

Hypogonadism is common in geriatric patients. If chronic anemia, sarcopenia, or osteoporosis are diagnosed, testosterone levels should be determined in geriatric settings.

**Supplementary Information:**

The online version of this article (10.1007/s00391-023-02235-7) contains supplementary material, which is available to authorized users.

## Abstract (de)

**Hintergrund:**

Zum Hypogonadismus bei sehr alten multimorbiden Männern gibt es kaum klinische Daten. Hypogonadismus kann zu Osteoporose, Anämie und Sarkopenie beitragen und ist daher ein relevantes Problem für geriatrische Patienten.

**Methoden:**

In diese Querschnittsstudie wurden 167 Männer im Alter von 65–96 Jahren (Mittelwert 81 ± 7 Jahre) aus einer Akutgeriatrie aufgenommen. Die anhand der Dual-Röntgen-Absorptiometrie (DXA) ermittelte Körperzusammensetzung sowie Knochendichtewerte, Handkraft, Multimorbidität, Polypharmazie und Laborwerte wurden den elektronischen Patientenakten entnommen.

**Ergebnisse:**

Bei 62 % (*n* = 104) der Studienteilnehmer wurde ein Hypogonadismus festgestellt. Von diesen wiesen 83 % eine klinische Manifestation des Hypogonadismus auf (Hypogonadismus in Kombination mit Anämie, Sarkopenie und/oder niedrigem T‑Score). Die Untergruppen zeigten eine Verteilung von 52 % primärem und 48 % sekundärem Hypogonadismus. Im Vergleich zu den eugonadalen Patienten wiesen die hypogonadalen Patienten eine geringere Handkraft (*p* = 0,031) und niedrigere Hämoglobinwerte (*p* = 0,043) auf, selbst nach Adjustierung für Alter, Body-Mass-Index und glomeruläre Filtrationsrate.

**Schlussfolgerung:**

Hypogonadismus tritt bei geriatrischen Patienten häufig auf. Wenn in geriatrischen Settings chronische Anämie, Sarkopenie oder Osteoporose diagnostiziert werden, sollten die Testosteronwerte bestimmt werden.

## Background

Testosterone deficiency in men is commonly referred to as hypogonadism [1, 2]. Starting from the third decade of life, there is an annual decline of approximately 0.4–2% in the free testosterone index [3–5]. The prevalence of hypogonadism exhibits significant variation across different studies and population groups. Specifically, in healthy outpatient groups of men the prevalence rates of hypogonadism range between 16% and 39% [6–8]. Iglesias et al. demonstrated an even higher prevalence of 53% in patients in an acute geriatric ward in Spain (*n* = 150, mean age 86 years) [9]. These examples show that men are often affected by hypogonadism. Hypogonadism can lead to various consequences, such as decreases in muscle mass and strength, energy levels, mood, libido, erectile function, and bone density [1, 2]. From a clinical perspective, the effects on sarcopenia, osteoporosis, and anemia are particularly relevant as they directly impact mobility, morbidity, and mortality in geriatric patients. Geriatric patients, as a vulnerable group characterized by factors, such as polypharmacy, multimorbidity, and limitations in mobility, require a special clinical focus.

Testosterone plays a crucial role in counteracting sarcopenia. It stimulates mesenchymal multipotent stem cells to differentiate into muscle cells while inhibiting adipogenesis. Additionally, testosterone promotes muscle stem cell replication, activates muscle protein synthesis, and inhibits protein degradation [10]. Clinical data of male kidney transplant recipients showed that decreasing testosterone levels are correlated with significant decline in handgrip strength (*n* = 144, mean age 72 years) [11]. Auyeung et al. described a positive association between testosterone and handgrip strength in a community-dwelling male cohort (*n* = 1489, mean age 72 years) [12]. Numerous studies have consistently shown that testosterone replacement increases lean body mass in men of older age [13].

In addition to its effects on the muscles, testosterone also plays a decisive role in maintaining bone mineral density (BMD). Along with estrogens, it stimulates osteoblast proliferation, partially mediated by cytokines and growth factors such as insulin-like growth factor 1 (IGF-1) [14]. Testosterone promotes bone mineralization and supports the maintenance of trabecular bone, while estrogens inhibit osteoclastogenesis. Additionally, testosterone is converted into estradiol via aromatization, which helps prevent bone loss. A recent review highlighted a prevalence of hypogonadism in men with osteopenia or fractures ranging from 7% to 58%, indicating a discrepancy in existing data and a potential diagnostic deficit for hypogonadism and osteoporosis in aging men [14]. Current data demonstrate that testosterone replacement significantly improves BMD, particularly in the lumbar spine region [15, 16]. Therefore, testosterone replacement is considered a treatment option for patients with osteoporosis and hypogonadism.

Chronic anemia in older men is a complex condition with multiple contributing factors. A clinical sign of hypogonadism can be mild anemia [17] as testosterone has the ability to increase erythropoiesis by stimulating erythropoietin production and expanding the number of erythropoietin-responsive cells in the bone marrow [18]. Roy et al. examined the effects of testosterone treatment in 126 men with low testosterone levels and unexplained anemia. After 12 months 54% of the treated patients showed an increase in hemoglobin (Hb) concentrations of at least 1 g/dl, compared to only 15% in the placebo group [19].

In summary, hypogonadism is a common condition that increases with age. Iglesias et al. found a correlation between hypogonadism and high mortality rates in geriatric patients [9]; however, there is a lack of studies investigating the prevalence of clinically evident hypogonadism characterized by clinical findings along with low testosterone levels in geriatric patients. Therefore, this study analyzed the difference between biochemical and clinically relevant hypogonadism, with a focus on sarcopenia, osteoporosis, and anemia in geriatric men.

## Methods

The methods part can be found in the supplementary file.

## Results

**Fig. 1:**
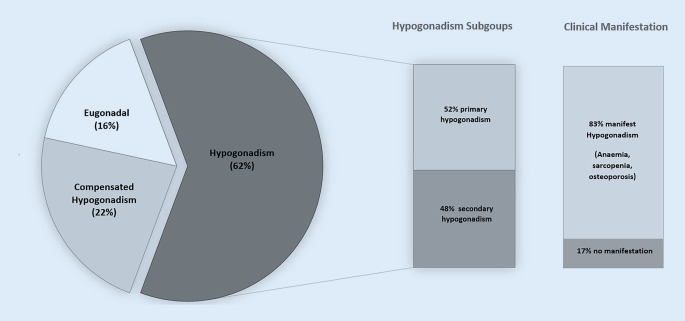
Distribution of hypogonadism and subgroups

**Fig. 2:**
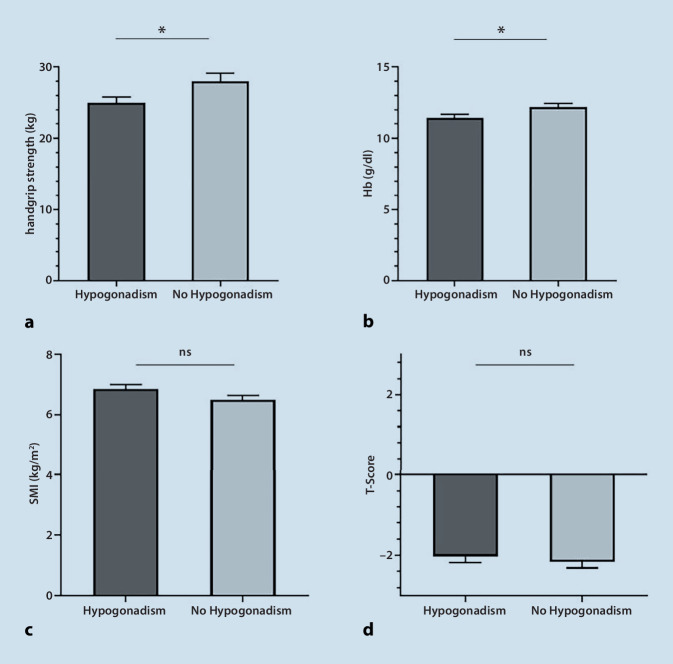
Manifest hypogonadism (**a-d**) **a** handgrip strength **b** Hemoglobin values (Hb) **c** skeletal mass index (SMI) **d** T-score is shown for the two groups hypogonadism and no hypogonadism; *ns* not significant, *p < 0.05

We enrolled a total of 167 men aged 65–96 years (mean 81 ± 7 years) from our acute geriatric ward. In this geriatric cohort, a prevalence of 62% (*n* = 104) for biochemical hypogonadism was observed. The two groups (hypogonadism vs. eugonadal) differed significantly in handgrip strength, probable sarcopenia and hemoglobin levels. Polypharmacy, characterized by an average use of 11 drugs per patient, and multimorbidity, with an average of 8 different diseases per patient, were observed in both groups. The hormone analysis showed a significant group difference for IGF‑I, but not for LH and FSH (Table 1). The subgroups showed a distribution of 52% primary and 48% secondary hypogonadism (Fig. 1). We could not find any subgroup differences regarding body mass index (BMI), handgrip strength, skeletal muscle mass index (SMI), hemoglobin level, GFR, T‑score, total number of comorbidities and polypharmacy (data not shown). Compensated hypogonadism (normal testosterone levels in combination with elevated LH levels) was present in 22% of all patients (Fig. 1. An extreme testosterone deficit (< 100 ng/dl) was present in 46 (44.2%) patients with hypogonadism (data not shown). Of all hypogonadal patients 83% (*n* = 86) presented with manifest hypogonadism (hypogonadism in combination with anemia 71%, sarcopenia 33% and/or low T‑score 46%) (Fig. 1). Differences in hemoglobin levels between the two groups (hypogonadism vs. no hypogonadism) remained significant after adjustment for age, BMI, GFR and IGF‑1. Differences in handgrip strength between the two groups (hypogonadism vs. no hypogonadism) remained significant after adjustment for age and BMI, but lost significance after adjusting for IGF‑1 (GFR did not significantly influence model 4). No significant differences were found for SMI and T‑score (Table 2, Fig. 2).

**Table 1:** Patients characteristics

Characteristic	Hypogonadism (*n* = 104)	Control (eugonadal) (*n* = 63)	*p*-value
*Age (years)*	80.1 ± 7.8	82.0 ± 6.4	0.119^a^
*Polypharmacy (number of drugs)*	11.0 ± 3.3	10.9 ± 3.2	0.799^a^
*Multimorbidity (number of diseases)*	7.6 ± 3.1	7.7 ± 3.2	0.847^a^
*BMI (kg/m*^*2*^*)*	25.8 ±5.1	24.7 ± 4.4	0.141^a^
*Fat mass (kg)*	23.8 ± 11.2	22.8 ± 8.8	0.572^a^
*SMI (kg/m*^*2*^*)*	6.9 ± 1.4	6.5 ± 1.0	0.088^a^
*Handgrip strength (kg)*	25.0 ± 8.9	28.0 ± 8.7	**0.031**^**a**^
*T‑score*	−2.0 ± 1.6	−2.2 ± 1.2	0.575^a^
*Sarcopenia*^*c*^	–	–	**0.017**^**b**^
Sarcopenia (*n* (%))	33 (32.4)	20 (33.3)	**–**
Probable sarcopenia (*n* (%))	24 (23.5)	4 (6.7)	**–**
No sarcopenia (*n* (%))	45 (44.1)	36 (60.0)	**–**
*Hemoglobin (g/dl)*	11.4 ± 2.4	12.2 ± 2.2	**0.043**^**a**^
*TSH (µU/ml)*	3.0 ± 4.5	2.2 ± 1.3	0.080^a^
*GFR (ml/min)*	69.6 ± 25.2	67.6 ± 22.5	0.538
*Albumin (g/dl)*	3.8 ± 0.5	3.8 ± 0.5	0.818
*25-OH-vitamin D (ng/ml)*	20.0 ± 36.2	19.4 ± 14.2	0.900
*IGF‑I (ng/ml)*	69.8 ± 36.3	96.1 ± 44.0	**<** **0.001**^**a**^
*Testosterone (ng/dl)*	116.8 ± 65.7	348.6 ± 128.7	**<** **0.001**^**a**^
*SHBG (nmol/l)*	55.4 ± 27.1	72.9 ± 27.7	**<** **0.001**^**a**^
*Free androgen index (%)*	8.9 ± 6.2	18.3 ± 8.0	**<** **0.001**^**a**^
*LH (U/l)*	14.1 ± 18.1	12.3 ± 9.0	0.444^a^
*FSH (U/l)*	14.7 ± 16.5	15.4 ± 12.0	0.814^a^

All measures are presented as mean ± SD unless otherwise noted

*BMI* body mass index, *SMI* skeletal muscle index, *TSH* thyroid-stimulating hormone, *GFR* glomerular filtration rate, *IGF‑I* insulin-like growth factor 1, *SHBG* sex hormone-binding globulin, *LH* luteinizing hormone, *FSH* follicle-stimulating hormone

^a^Student’s t‑test

^b^χ^2^-test

^c^5 patients are missing DXA measurements

**Table 2:** Multiple linear regression analysis

	Independent variable: hypogonadism			
Dependent variable	Beta	95% CI	SE	*p*-value
*Hemoglobin*				
Model 1	−0.16	−1.49–−0.24	0.37	0.043*
Model 2	−0.17	−1.56–−0.08	0.37	0.029*
Model 3	−0.18	−1.63–−0.14	0.37	0.020*
Model 4	−0.29	−1.76–−0.14	0.41	0.022*
*T‑score*				
Model 1	0.04	−0.33–0.60	0.24	0.575
Model 2	0.05	−0.33–0.61	0.24	0.566
Model 3	0.03	−0.39–0.55	0.24	0.736
Model 4	0.02	−0.49–0.58	0.27	0.865
*Handgrip strength*				
Model 1	−0.17	−5.90–−0.29	1.42	0.031*
Model 2	−0.18	−6.18–−0.59	1.42	0.018*
Model 3	−0.18	−6.23–−0.56	1.43	0.019*
Model 4	−0.11	−5.17–1.01	1.56	0.185
*SMI*				
Model 1	0.14	−0.06–0.79	0.21	0.088
Model 2	0.13	−0.08–0.77	0.22	0.113
Model 3	0.06	−0.14–0.45	0.15	0.292
Model 4	0.08	−0.10–0.51	0.16	0.185

*Model 1* unadjusted, *model 2* adjusted for age, *model 3* adjusted for age and body mass index (BMI), *model 4* adjusted for age, BMI, glomerular filtration rate (GFR) and insulin-like growth factor 1 (IGF-1)*, SMI* skeletal muscle index, *beta* standardized regression coefficient beta ,*CI* confidence interval, *SE* standard error

## Discussion

The study revealed a high prevalence of hypogonadism, with 62% (*n* = 104) of geriatric men (mean age 81 years) admitted to a geriatric ward being affected. Among them, a significant majority (83%, *n* = 86) exhibited manifest hypogonadism, characterized by the coexistence of hypogonadism with anemia, sarcopenia, and/or low T‑score. The complete study cohort exhibited multimorbidity, with an average of eight diseases per patient, and was accompanied by polypharmacy, both common characteristics of geriatric cohorts.

### Hypogonadism subgroups

Regarding the subgroups of hypogonadism, the findings showed a distribution of 52% primary and 48% secondary hypogonadism. The European male ageing study, which included 3369 men aged 40–79 years from a single community, reported an increased prevalence of primary and compensated hypogonadism (normal testosterone with elevated LH) with advancing age [20]. Furthermore, this study found associations between both primary and secondary hypogonadism and various comorbidities, such as heart conditions, high blood pressure, cancer, bronchitis, asthma, peptic ulcer, epilepsy, and diabetes [20]; however, data on whether different hypogonadism subgroups lead to distinct clinical consequences are currently lacking. Our study did not reveal any subgroup differences in terms of BMI, handgrip strength, SMI, hemoglobin levels, GFR, T‑score, number of comorbidities, or polypharmacy.

Another relevant subgroup worth considering is compensated hypogonadism, characterized by normal testosterone levels combined with elevated LH/FSH levels. A recent review indicated that compensated hypogonadism is common, affecting approximately 9% of aging men in the general population [21]. In our specific setting, we observed that 22% of participants exhibited compensated hypogonadism, suggesting a higher proportion compared to the general population within the geriatric context. Longitudinal data exposed that compensated hypogonadism is a sign for poor health and increased cardiovascular mortality [21]; however, information on the therapeutic implications of compensated hypogonadism is currently lacking.

### Hypogonadism and sarcopenia

The prevalence of sarcopenia in men over 65 years old is approximately 6% [22]. Our assessment showed that handgrip strength was reduced by an average of 3kg compared to the eugonadal group. These findings align with existing literature demonstrating a negative correlation between handgrip strength and testosterone levels in untreated patients [11, 12]. Interventional studies have indicated that testosterone replacement in older men can result in increased muscle strength [12, 23]. The impact of this correlation was modulated by IGF‑1, highlighting the interplay between these factors. Previous studies have established a connection between testosterone and IGF‑1 in the endocrine system. Testosterone can influence the production of IGF‑1, and in turn IGF‑1 levels can affect testosterone secretion [24].

We did not observe any significant correlations for SMI, suggesting that muscle function, as represented by handgrip strength, may be affected earlier than muscle mass. This is a common phenomenon in sarcopenia, where muscle strength tends to decline more rapidly and earlier than muscle mass [25]; however, this emphasizes the importance of recognizing muscle mass and function as separate entities. A study by Van den Beld et al. supported this notion, demonstrating that testosterone was associated with muscle strength but not with muscle mass in a cohort of healthy, independently living older men with a mean age of 78 years [26]. In interventional studies, testosterone treatment has generally shown improvement in lean body mass [27–30]; however, there is a lack of longitudinal data specifically examining testosterone treatment in geriatric men. Therefore, further studies utilizing the EWGSOP2 definition for sarcopenia are needed to provide more comprehensive insights.

### Hypogonadism and osteoporosis

The prevalence of osteoporosis in men in the age group 70–80 years is approximately 4% [31].

We did not find any significant differences in T‑scores between the hypogonadal and eugonadal groups. Interestingly, both groups had a mean T‑score of −2.0, indicating lower bone density. It is important to consider that the methodology of dual-energy X‑ray absorptiometry (DXA) itself may contribute to this finding. Research conducted by our group has shown that DXA loses sensitivity with increasing age, possibly due to confounders, such as aortic calcifications, incorrect positioning, spondylophytes and hip implants [32]. In contrast, peripheral quantitative computed tomography (pQCT) offers several advantages over DXA for assessing BMD in geriatric patients. It enables volumetric assessment, structural analysis, reliable performance in older age, and improved fracture prediction [32].

However, there are measurable effects on bone density under testosterone treatment. Placebo-controlled studies have demonstrated that after 1 year of testosterone replacement, there is an increase in hip and spine BMD [15, 16]. Specifically, there was an improvement in bone strength in the trabecular zone [16]. In a meta-analysis conducted by Isidori et al. involving 1083 participants, it was found that lumbar spine bone density improved by 4% and fat mass was reduced by 6% compared to the placebo group after a minimum of 12–36 months of testosterone replacement [32]; however, in osteoporosis research the likelihood of fractures is a crucial outcome of interest. Therefore, it is important to conduct further studies that not only focus on changes in bone composition but specifically address the incidence of major osteoporotic fractures allied to hypogonadism.

### Hypogonadism and anemia

Anemia is a common association with hypogonadism, as testosterone plays a crucial role in stimulating erythropoiesis [33]. Around 30% of all anemia cases in geriatric patients are of unknown etiology and diminished testosterone levels could be a major cause [33]. In our data hemoglobin concentrations differed significantly between hypogonadal and eugonadal patients even after adjustment for GFR. These findings are consistent with the results of Lee et al., who analyzed testosterone and hemoglobin levels in a matched cohort of 444 hypogonadal and 7924 eugonadal men with a mean age of 51 years [34]. Even in this relatively young outpatient group, the hypogonadal participants exhibited lower mean hemoglobin concentrations and a higher incidence of anemia [34]. The relative risk of anemia in the hypogonadal group was 2.4 compared to the eugonadal group [34]. Zhang et al. demonstrated that hemoglobin values significantly increased after long-term (54 weeks) testosterone replacement [35]. Polycythemia, a contraindication of testosterone substitution, occurred less frequently under transdermal replacement [36, 37]. In summary, in geriatric men with unclear and/or unresponsive chronic anemia, the determination of testosterone should be considered.

## Strength and limitations

The strengths of our study include the recruitment of a consecutively enrolled high-risk geriatric patient population, with a mean age of 81 years, and the utilization of standardized sarcopenia assessment based on the revised EWGSOP2 criteria using DXA. Considering the challenges associated with conducting clinical studies involving geriatric patients, our study boasts a relatively large sample size. To the best of our knowledge, we are the first to demonstrate the prevalence of manifest hypogonadism in geriatric men, highlighting its clinical significance in relation to anemia, osteoporosis, and sarcopenia. It is important to note that hypogonadism is not merely a laboratory diagnosis but holds significant clinical relevance for this patient group.

However, our study does have several limitations. Firstly, it focused exclusively on hospitalized patients, which introduces the possibility that the observed prevalence of hypogonadism may have been influenced by the acute illnesses. Secondly, the cross-sectional design of our study prevents us from establishing a definitive causal relationship between hypogonadism and the observed outcomes.

## Practical conclusion

Hypogonadism is common in geriatric patients. Therefore, if unexplained anemia, sarcopenia, or osteoporosis are diagnosed in geriatric men, testosterone levels should be determined. Hormone treatment might be considered after careful evaluation of risks and benefits and after exclusion of contraindications.

### Supplementary Information

Methods used for the study

## Abbreviations

BMD: Bone mineral density
CV: Coefficients of variation
DXA: Dual-energy X‑ray absorptiometry
EWGSOP: European Working Group on Sarcopenia in Older People
FAI: Free androgen index
FSH: Follicle stimulating hormone
GFR: Glomerular filtration rate
Hb: Hemoglobin
LH: Luteinizing hormone
pQCT: Peripheral quantitative computed tomography
SHBG: Sex hormone-binding globulin
SMI: Skeletal muscle mass index

## Declarations

All procedures performed in studies involving human participants or on human tissue were in accordance with the ethical standards of the institutional and/or national research committee and with the 1975 Helsinki declaration and its later amendments or comparable ethical standards. Since this study was retrospective in nature, it did not require informed consent from the participants. The study protocol received approval from the Ethical Review Committee of Ludwig-Maximilians-University under Ethical Vote No. 22‑0305.
