# Supplementary material for: Hypogonadism is frequent in very old men with multimorbidity and is associated with anemia and sarcopenia
Source: Z Gerontol Geriatr. 2023 Sep 6;57(1):43–9. doi: 10.1007/s00391-023-02235-7 (PMC10827893; doi:10.1007/s00391-023-02235-7)
Supplement: Supplementary file 1 — Methods used for the study [file 391_2023_2235_MOESM1_ESM.pdf]

Supplementary Part to

Hypogonadism is frequent in very old men with multimorbidity and is associated with anemia and sarcopenia

Sabine Schluessel[1] • Martin Bidlingmaier, MD[2] •Sebastian Martini, MD[1] • Martin Reincke, MD[2]• Nicole Reisch, MD[2] • Anna Schaupp, MD[1] • Günter Stalla, MD[3] •Daniel Teupser, MD[4]• Ralf Schmidmaier, MD[2]• Michael Drey, MD[1]

[1] Medizinische Klinik und Poliklinik IV, Department of Geriatrics, LMU Klinikum, Ludwig-Maximilians-Universität München, Munich, Germany.

[2] Medizinische Klinik und Poliklinik IV, Department of Endocrinology, LMU Klinikum, Ludwig-Maximilians-Universität München, Munich, Germany.

[3] Medicover Neuroendocrinology, Munich Germany.

[4] Institute of Laboratory Medicine, LMU Klinikum, Ludwig-Maximilians-Universität München, Munich, Germany.

Design and Study Population

Patient data was obtained cross-sectional from the acute geriatric ward of the LMU University Hospital Munich, Germany in a single-centre study between January 2018 and October 2021. Data from patients older than 65 years was taken if their electronic routine clinical data file contained the necessary data including body composition, bone mineral density, handgrip strength and endocrinological laboratory measures (testosterone, SHBG, LH and FSH). Men with androgen deprivation therapy were excluded. The protocol was approved by the ethical review committee of the Ludwig-Maximilians-University (Ethical vote no. 22-0305).

Sarcopenia Definition

According to the revised consensus definition of the European Working Group on Sarcopenia in Older People (EWGSOP2), sarcopenia is defined by low muscle strength and low muscle mass (20). Muscle mass assessed by a skeletal muscle mass index (SMI) below 7.0 kg/m^2 by dual-energy X-ray absorptiometry (DXA) and muscle strength measured by handgrip strength below 27kg indicate sarcopenia. Probable sarcopenia was defined as normal SMI (≥ 7.0 kg/m^2) in combination with reduced handgrip strength (<27kg).

Definition of hypogonadism

Hypogonadism was diagnosed if total testosterone levels were below the cut-off-value of 231 ng/dl (= 8nmol/l) (1, 2). Hypogonadism subgroups were divided into primary (LH elevated) and secondary (LH normal or reduced) hypogonadism. Manifest hypogonadism was defined by additional anaemia (Hemoglobin < 13g/dl), low T-Score (T-Score< -2.5) and/ or sarcopenia. Compensated hypogonadism is specified as normal testosterone levels in combination with elevated LH levels.

Measurement of muscle strength

Handgrip strength was measured using a hydraulic handheld dynamometer (JAMAR, Los Angeles, CA). Three measurements were taken from each side, and the highest value was used for diagnosis and taken for analysis. Values below 27 kg for men were considered pathologically reduced handgrip strength (20).

Measurement of body composition and bone mineral density

Body composition was measured by dual-energy X-ray absorptiometry (DXA). The appendicular skeletal muscle mass was obtained as the sum of appendicular lean mass (ALM) of both arms and legs. The SMI was calculated by dividing the ALM by height in meters squared (kg/m^2). SMI values below 7.0 kg/m2 were considered pathologically reduced SMI. The T-Score was also determined by DXA investigating spine and hip. The lowest value of spine, femoral neck or total femur was used for analysis. The BMD of the lumbar spine was analysed according to the advices of the ISCD official positions, excluding vertebrae that were clearly abnormal or had more than a T-score difference to adjacent vertebrae. In this case the T-score or BMD of the lumbar spine vertebrae 1-4 was computed with the remaining vertebrae (21).

Measurement of polypharmacy and multimorbidity

Polypharmacy and multimorbidity were retrospectively assessed using the discharge medical records. The count of medications was based on the information provided in the discharge medication list, excluding supplements and vitamins. Multimorbidity was determined by reviewing the diagnosis list. Pre-existing conditions that were no longer present at the time of hospitalization were not included in the assessment.

Laboratory measurements

All study subject underwent fasting blood sampling between 8 and 10 a.m. Biochemical evaluation was performed at the Endocrine Laboratory of the University Hospital Munich (Martin Bidlingmaier, KUM, Germany). Serum testosterone and sex hormone-binding globulin (SHBG) levels were determined using the IDS-iSYS chemiluminescence immunoassays (Immunodiagnostic Systems, (IDS) Ltd., Boldon, England, UK). In our hands, intra-assay coefficients of variation (CV) at various concentrations ranged from 2.2-5.2% for testosterone and 1.9-2.6% for SHBG. The between-assay CVs ranged from 2.3-18.6% for testosterone, and 2.2-7.8% for SHBGThe free androgen index (FAI) was calculated from the molar concentrations of testosterone and SHBG according to the following formula: Testosterone (nmol/L) / SHBG (nmol/L) x 100.

Serum IGF-I levels were measured using the IDS-iSYS chemiluminescence immunoassays (Immunodiagnostic Systems, (IDS) Ltd., Boldon, England, UK), calibrated against the latest recombinant standards (02/254 for IGF-I). In our hands, intra-assay CVs were between 1.1 and 2.0% at concentrations between 87 and 733 ng/mL, whereas inter-assay CVs ranged from 2.7 to 10.0% at concentrations between 68 and 490 ng/mL. Extensive validation data as well as age-adjusted reference intervals for the assays used have been published (22).

Serum concentrations of follicle stimulating hormone (FSH) and luteinizing hormone (LH) were determined using specific automated electrochemiluminescence immunoassays (Roche Diagnostics, Mannheim, Germany). Within and between assay CVs were <15%.

Statistical analysis

All metric and normally distributed variables are reported as mean and standard deviation, categorical variables as frequency and percentage. Comparing groups, Student's T-test in case of metric variables, and Chi-square-test for categorical variables was used. Multiple linear regression analyses were performed for the dependent variable hemoglobin, T-Score, handgrip strength and SMI. All statistical calculations were performed using software package SPSS (v27.0, SPSS Inc, Chicago, IL, USA). *P* values < 0.05 were considered statistically significant. Graph Pad Prism 8.3.0. for Windows (GraphPad Software, San Diego, CA, USA) was utilized to visualize the data.
